# Allelic gene expression imbalance of bovine *IGF2*, *LEP* and *CCL2* genes in liver, kidney and pituitary

**DOI:** 10.1007/s11033-012-2161-3

**Published:** 2012-11-25

**Authors:** R. Olbromski, E. Siadkowska, B. Żelazowska, L. Zwierzchowski

**Affiliations:** Department of Molecular Biology, Institute of Genetics and Animal Breeding, Polish Academy of Sciences (IGAB PAS), Jastrzębiec, 05-552 Magdalenka, Poland

**Keywords:** Allelic expression imbalance, Transcription regulation, Holstein–Friesian cattle, Gene expression

## Abstract

Allelic expression imbalance (AEI) is an important genetic factor being the cause of differences in phenotypic traits that can be heritable. Studying AEI can be useful in searching for factors that modulate gene expression and help to understand molecular mechanisms underlying phenotypic changes. Although it was commonly recognized in many species and we know many genes show allelic expression imbalance, this phenomena was not studied on a larger scale in cattle. Using the pyrosequencing method we analyzed a set of 29 bovine genes in order to find those that have preferential allelic expression. The study was conducted in three tissues: liver, pituitary and kindey. Out of the studied group of genes 3 of them—*LEP* (leptin), *IGF2* (insulin-like growth factor 2), *CCL2* (chemokine C–C motif ligand 2) showed allelic expression imbalance.

**Electronic supplementary material:**

The online version of this article (doi:10.1007/s11033-012-2161-3) contains supplementary material, which is available to authorized users.

## Introduction

Allelic expression imbalance (AEI) is a phenomena where one of the allelic transcripts is overrepresented, relative to the other one in a gene transcript pool. Sources of such difference can be multiple—nevertheless, it is presumed that if AEI is present, then it must be connected with the occurrence of minimum one *cis*-regulatory element in the regulatory sequences of the gene [1].

AEI is a subject of increasing interest, and it appears to be more common than previously thought and could account for phenotypic differences [2]. Depending on the studies, the fraction of genes showing bias between alleles in transcription ranges from 5 to 54 % [3]. Usually the skew in allelic expression is one-directional—that is, there is a preferably expressed allele in the studied population [4]. The disproportion in allelic expression can be various—from a few percent variation, up to monoallelic expression. Further studies showed that allelic expression can be tissue-specific but not gender-specific. Interestingly, experiments conducted on the basis of human global AEI analysis, showed that genes harboring differences in allelic expression in humans were more likely to show allelic imbalance in mice [1].

AEI has been studied in various species: human [4–7], mouse [1, 8], pig [9], yeast [10], maize [11], *A. thaliana* [12], chicken [13]. So far only a few studies have been conducted on cattle, usually analyzing AEI in single genes [14–18]. Because AEI is a consequence of the presenece of one or more *cis*-regulatory factors, it could be used as a tool to indicate them [3]. Such attempts were already made mainly in the subject of human disease susceptibility [2, 19]. The same schematic can be used in other species to find factors significantly modulating expression of genes important, for example in livestock breeding. A factor that causes variation in allelic expression, will surely affect the overall expression of a gene. One of the methods to study AEI is following allelic expression using SNPs in the transcribed region of a gene. Often, the polymorphic change in the coding region of a gene can be in likage disequilibrium with the causative *cis*-regulatory element [1]. As significant number of AEI differences can be hereditary [5], the use of this phenomena in animal breeding seems to be justified.

The objective of this study was to analyze allelic expression of 29 randomly chosen genes in bovine liver, kidney and pituitary. This is the first study that analyses expression of a larger group of bovine genes in search for AEI.

## Materials and methods

### Animals

The study was conducted on 40 unrelated Polish Holstein–Friesian bulls from the local Institute farm. The bulls were fed hay and corn silage. Roughage was balanced with concentrate in accordance to animal age. From the 11 month of age the animals were fed 2 kg of roughage. They were kept in free-stalls. The bulls were slaughtered at the age of 12 months.

All procedures involving animals were performed in accordance with the Guiding Principles for the Care and Use of Research Animals and were approved by the Local Ethics Commission (Warsaw Agricultural University; Permission No. 23/2008).

### DNA isolation and genotyping

Ten ml of blood was withdrawn from each bull on K_2_EDTA and stored in −80 °C upon further use. Genomic DNA was isolated according to Kanai et al. [20].

To analyze AEI the allelelic transcripts had to be differentiated from one another. This was done by choosing a single nucleotide polymorphism located in the coding region of a gene (cSNP) so that it could be expressed in the transcript pool. Therefore cSNPs were selected in all 29 genes under study.

The genes were chosen from publications that describe polymorphisms in bovine genes—optimally in HF population. All of the selected genes have various functions: metabolic (*LEP, SCD, PI, PSAP, FASN, NOS2, PPARGC1A*), immune system (e.g. *CD14, CCL2, PVRL2, NOD2, CXCR2*), hormones and hormone receptors (e.g. *ERB, GH, GHR, PRL*), transcription factors (e.g. *MED28, PIT1, STAT1*), cell structure and proliferation (e.g. *CDH1, IBSP, ITGB5*). Therefore polymorphisms in these genes could have a crucial effect on the animal phenotype. Most of these genes were described in publications as potential markers in cattle production. Thus finding variants that modulate expression of these genes may be very useful in breeding.

Each individual from the group of 40 HF bulls was genotyped and heterozygotes were taken for the allelic expression analysis as only in these samples was it possible to quantify the allelic transcript pools.

Genotyping was performed using the PCR–RFLP or forced-PCR–RFLP methods with the genomic DNA isolated from blood as a template. Forced-RFLP gave us the possibility to genotype SNPs not placed in a cutting site for a restriction enzyme. One of the starters was placed just before the mutation and one of the nucleotides was altered near the starter 3′-end, creating a sequence for the enzyme to recognize.

Primer sequences were taken from publications or designed using Primer3 software (http://frodo.wi.mit.edu/primer3/). Restriction maps were obtained using NEBcutter V2.0 software (http://tools.neb.com/NEBcutter2/). Genotyped cSNPs and primer sequences are listed in Table 1. All 40 animals were genotyped for each SNP included in the study.

**Table 1:** Genes analyzed and SNPs used for AEI survey in tissues of Holstein–Friesian cattle, along with restriction enzymes and primers used for RFLP and annealing temperatures

Gene name	GenBank	cSNP	Restriction enzyme	SNP reference^#^	Forward primer 5′–3′	Annealing temperature (°C)
					Reverse primer 5′–3′	
*CDH1*	NC_007316.4	c.2102 C/T	*Mse*I	[21]	CAATCCCCTCATTTTTGTTG	52
					GAACTTGCAATCCTGCTTTA	
*PVRL2*	NC_007316.4	c.392 G/A	*Hha*I	[21]	CACCCTGCTCACCTATGACT	54
					ATCTTCCACCCCCAGTATG	
*PPARGC1A*	NC_007304.4	c.1209 T/C	*Pag*I	[22]	TTAGTACATCACAGGAGCTTCA	55
					CGGTCTCTCTCAGGTAGCAC	
*FASN*	NC_007317.4	g.16024 A/G	*Hha*I	[23]*	CTACCAAGCCAGGCAGGTC	60
					GCCATTGTACTTGGGCTTGT	
*CXCR2*	NC_007300.4	g.7148 G/C	*Bae*GI	[24]	GGTGCCAATACAACGAAATG	58
					AGCAGAGCAGGAAGACGAG	
*CD14*	NC_007305.4	g.1105 A/G	*Bce*AI	[25]	GTGCTACCCGATGTGTCTG	54
					TCATTCCTCTTCCCTCTCTTC	
*MED28*	NC_007304.4	g.5413 C/T	*Mse*I	[26]	CAGGCATCTTTCGTGGAA	60
					CTCAGGTTTGCTTCATTGGT	
*FAM13A1*	NC_007304.4	g.79871 C/A	*Ava*II	[26]	ATACATCTCCACGCCCAAAT	58
					GCTCATCACAGAATCACACCT	
*IBSP*	NC_007304.4	c.802 A/G	*Aci*I	[26]	AAACCTACAACCCCACACCA	60
					AATTGTCCCCACGAGGATCT	
*PIT1*	NC_007299.4	g.15637 G/A	*Hinf*I	[27]*	CAATGAGAAAGTTGGTGC	55
					TCTGCATTCGAGATGCTC	
*GHR*	NC_007318.4	g.173395 A/G	*Alu*I	[28]*	CTATGGCATGATTTTGTTCAG	55
					GCTAACTTCATCGTGGACAAC	
*PRL*	NC_007324.4	g.7550 A/G	*Rsa*I	[29]*	CGAGTCCTTATGAGCTTGATTCTT	48
					GCCTTCCAGAAGTCGTTTGTTTTC	
*IGF2*	NC_007330.4	g.24507 G/T	*Hae*III	[55]*	AATCCCTGTACCGTCCTGTC	56
					TTTGCTTTTCTGTGTTTGCT	
*GH*	AC_000176.1	c.440 C/G	*Alu*I	[30]*	CCGTGTCTATGAGAAGC	60
					GTTCTTGAGCAGCGCGT	
*ERB*	NC_007308.4	g.46616 C/G	*Ear*I	[31]*	CTCTTGGGGGAGTAGACA	60
					CTACTACAACGACCGCATC	
*PRNP*	NC_007311.4	g.18113 A/G	*Ssp*I	[32]	CAAAATTAGGTCCTTGGTTTCTG	58
					CCACAAAGTGCAAGCCAGTA	
*ITGB5*	NC_007299.4	g.115160 C/T	*Bcc*I	[33]	CAACCCTGTGTGTTCGAATG	55
					GCTTGCCGGAAGGTCTCT	
*NOS2*	NC_007317.4	c.140 C/T^a^	*Ava*I	GenBank AF333248	TGCAGTGAGTTGAAGACTGAGA	58
					ATGCAGGGTCTCGACAAGAG	
*STAT1*	NC_007300.4	c.3132 C/T^b^	*Pag*I	[34]	GCCTCAAGTTTGCCAGTGGC	58
					GGCTCCCTTGATAGAACTGT	
*TNFα*	NC_007324.4	g.1844 C/T	*Rsa*I	[35]	CATCCTGTCTGCCATCAAGA	56
					GGCGATGATCCCAAAGTAGA	
*LEP*	NC_007302.4	g.12140 C/T	*Kpn*2I	[36]*	ATGCGCTGTGGACCCCTGTATC	58
					TGGTGTCATCCTGGACCTTCC	
*SCD*	AC_000183.1	c.3290 C/T	*Hha*I	[37]	TCTTCCTCTGTCTGGGTCAG	53
					AACCTGCCTTTGCTTCTTGT	
*PI*	NC_007319.4	c.989 C/T	*Rsa*I	[38]	ATAAACCAAAGTGTGAGAGCAG	58
					CCCTATCGCTGAAGACCTC	
*CCL2*	NC_007317.4	c.249 C/T	*Nla*III	[39]	CCTCGAAGAACATTCAGGTCA	58
					GTAGATGATGGGGTTGATGC	
*ODC1*	NC_007309.4	g.5464 G/A	*Msp*I	[40]	TCCCTTGATGACCAACTGCT	58
					TAACTGCGAGCGTGAAAGC	
*TGFβ1*	NC_007316.4	g.3960 C/T	*Dde*I	[41]	AAGAGGTGGAAACAAACTCAGA	58
					GAGAGAGCAACACAGGTTCG	
*IL10RB*	NC_007299.4	c.608 C/T	*EcoR*I	[41]	AAATGATGTCCCTTCACTGC	54
					TCAGAAAGAAACCCTCGAATT	
*PSAP*	NC_007329.4	g.22079 A/G	*Bcc*I	[40]	GGGTTGAGTGGTTCAGTTTG	55
					TACAGGAGGAAGGGGATGTT	
*NOD2*	NC_007316.5	g.27861 A/T	*Pvu*II	[42]	AATTGAGAAACTCAGCCAGC^c^	55
					GTGCCAGAACAAAGGTGAC	

Primers used in forced-RFLP have the substituted nucleotides underlined. References marked with an asterisk indicate studies from which PCR primer sequences were used. Otherwise the primers were designed for the purpose of this study

^a^Nucleotide position according to GenBank accession number AF333248

^b^Nucleotide position according to GenBank accession number BC151378

^c^The starter with a modified nucleotide for forced-RFLP spans from g.27841 to g.27860, with G replacing C at position g.27859

PCR was performed using the following mix (10 μl): 0.5 μl 10 μM forward and reverse primers, 5 μl REDTaq^®^ ReadyMix™ (Sigma-Aldrich, Munich, Germany), and approximately 100 ng genomic DNA. The PCR reactions were carried out in a MJ TETRAD thermocycler (Bio-Rad, Hercules, USA) at temperatures and cycling conditions optimal for each gene, previously experimentally established.

The PCR products were digested with a respective restriction endonuclease (see Table 1). The restriction products were separated by electrophoresis in 2 % agarose gel (Sigma-Aldrich) with ethidium bromide in TBE buffer. Bands were visualized and documented by the Molecular Imager System FX (Bio-Rad, Hercules, USA).

### Tissue collection, RNA isolation and reverse transcription

Liver, kidney and pituitary samples were collected immediately after slaughter, flash-frozen in liquid nitrogen and stored at −80 °C upon further use. The tissues were disrupted and homogenized using the FastPrep24 instrument (MP Biomedicals, Solon, OH, USA).

RNA isolation was conducted using the Nucleospin RNA II Kit (Machrey-Nagel GmbH, Duren, Germany), according to the manufacturer’s manual. During the isolation RNase-free DNase treatment has been performed to be sure no residual genomic DNA is carried over to the final RNA isolate. The purified RNA was quantified using Nanodrop^®^ spectrophotometer (Wilmingtion, USA). The quality of RNA was analyzed on a 1.5 % agarose gel. Two μg of RNA was reverse transcribed using the M-MLV reverse transcriptase and oligoT-nucleotides (PROMEGA, Madison, USA) according to the manufacturer’s manual.

### PCR, template preparation and pyrosequencing

Prior to the pyrosequencing reaction the PCR product had to be labeled with biotin. Therefore, we used a set of three primers: two amplification primers (forward and reverse) specific for the locus of interest, and a universal M13 phage-derived primer labeled with biotin at its 5′-end (5′-biotin-CGCCAGGGTTTTCCCAGTCACGAC-3′). One of the amplification primers (depending on the sequencing direction) had an additional M13 sequence at its 5′-end thus creating a target sequence in the amplicon for the labeled M13 primer to attach. This approach made it possible to simultaneously amplify the product and label it with biotin. The primers were designed using PyroMarkQ24 software (Qiagen GmbH, Nordrhein-Westfallen, Germany), and ordered in Institute of Biochemistry and Biophysics, PAS, Warsaw, Poland.

PCR was performed using the following mix (30 μl): 0.1 μl or 0.9 μl of 10 μM forward or reverse primers (depending on the direction of sequencing), 0.8 μl of 10 μM 5′-biotinylated M13 primer, 0.75 U of HotStarTaq^®^ DNA Polymerase (Qiagen GmbH, Nordrhein-Westfallen, Germany), 3.75 μl of 10 × polymerase buffer, dNTPs each at concentration of 2.5 mM, 1.5 mM final concentration of MgCl_2_, and approximately 300 ng cDNA. The PCR was carried out in a MJ TETRAD thermocycler (Bio-Rad, Hercules, USA) at temperatures and cycling conditions optimal for each gene, previously experimentally established. For primer sequences see Supplementary Table 1. After amplification, 10 μl of each sample was checked for product specificity in a 2 % agarose gel. Next, the biotinylated amplicon was conjugated with streptavidin and vortexed for 10 min at 1,300 rpm. After vortexing amplified DNA was separated from the PCR mix using the PyroMark Q24 Vacuum Workstation (Qiagen GmbH, Nordrhein-Westfallen, Germany). Post to immobilization on a membrane by the vacuum, the amplicon was washed in 70 % ethanol for 5 s, Denaturation Solution for 5 s, and in Wash Buffer for 10 s. Then the vacuum was stopped and the single-stranded amplicon was released to the Annealing Buffer with sequencing primer placed in a pyrosequencing plate.

The samples on the plate were incubated at 80 °C for 2 min and then after cooling to room temperature placed in the PyroMark Q24 instrument (Qiagen GmbH, Nordrhein-Westfallen, Germany) for further analysis. The amount of used Enzyme mix, Substrate, and dNTPs was calculated by the manufacturer’s software. The ratio of allelic transcripts was obtained by comparing the percentage of incorporated nucleotides at an analyzed cSNP locus.

**Fig. 1:**
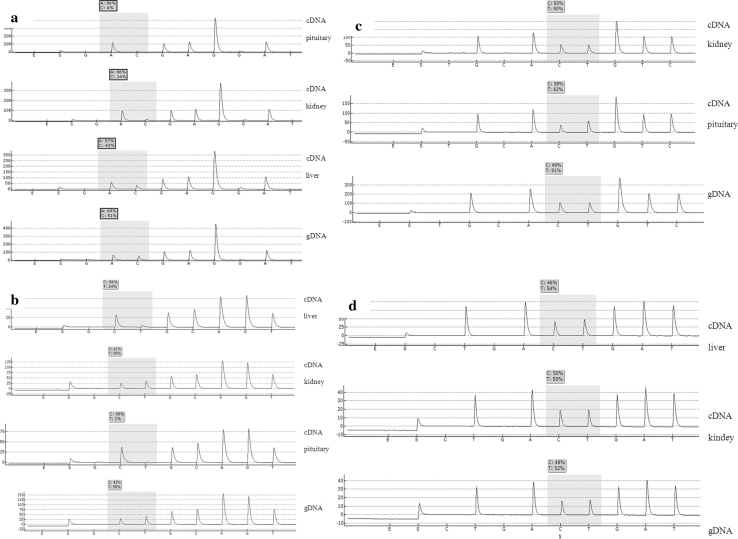
Sample pyrograms of the bovine *IGF2* (**a**)*, LEP* (**b**)*, CCL2* (**c**), *PI* (**d**) genes. In all figures the SNP used as a maker is shaded; above are the proportions of the incorporated nucleotides

Each gene had to have a reference sample showing a balance between transcripts. For this purpose we pyrosequenced samples amplified from genomic DNA (gDNA) from heterozygous animals. Heterozygous gDNA should theoretically show a perfect balance between gene copies. Therefore all cDNA sample ratios were standarized according to gDNA ratio. We assumed 60:40 or 40:60 thresholds for a gene to be considered as imbalanced. Figure 1 shows sample pyrograms of the bovine *IGF2, LEP, CCL2* and *PI* presenting cDNA samples and gDNA samples used as balanced reference.

### Statistical analysis

To assess whether the differences in AEI between gDNA and cDNA in a tissue are significant we used a two-tailed *t* test. The percentage representation of alleles in the transcript pool was changed into allelic ratios by dividing the percentage of one allele by the other. For each cDNA sample, its allelic ratio was divided by the mean allelic ratio of gDNA for a certain gene. This way the effect of deviation in gDNA ratio (maximum detected 8 %, on average 3 %) could be neutralized. The skew is probably a consequence of the reaction nature. Nucleotide incorporation at mSNP position can give small deviations in gDNA allelic ratio. Although small, these differences had to be included in calculations, as not to omit small differences in cDNA allelic ratio, exceeding the 60:40 threshold. All imbalances in a studied tissue for a gene were taken as one, neglecting the directional character of the imbalance; the ratio was calculated by dividing the higher percentage by the lower one. This way we could see whether the whole imbalance is really significant omitting the bidirectional imbalance bias.

### Promoter analysis

To find the possible causative factors for AEI, gene regulatory sequences were analyzed for SNPs in putative transcription factor binding sites and the presence of CpG islands as markers of probable methylation patterns.

*LEP*, *IGF2* and *CCL2* genes 5′-flanking regions were analyzed for the presence of CpG islands using UCSC Genome Browser (http://genome.ucsc.edu/).

SNPs in 5′-flanking regions were imported from dbSNP using Ensembl Biomart online software (http://www.ensembl.org). Putative transcription factor binding site analysis was conducted using TESS software (http://www.cbil.upenn.edu/cgi-bin/tess/tess) and Transfac 7.0 database (http://www.gene-regulation.com/cgi-bin/pub/databases/transfac/search.cgi).

In *LEP* we used the bovine promoter sequence (GenBank accession number AJ571671). For *CCL2* analysis we used a 3.0 kb fragment upstream to the transcription start site (TSS). To find the sequences of *IGF2* promoters we compared parts of human *IGF2* promoter sequences (EP17071, EP28010, EP17072, EP28009; 500 bp upstream to TSS from each sequence) downloaded from The Eucariotic Promoter Database (http://epd.vital-it.ch/) to the bovine INS-IGF2 locus sequence (GenBank accession number EU518675) using BLAST (http://blast.ncbi.nlm.nih.gov/). Sequences showing similarity were used for further analysis.

## Results

### Genotyping

We chose genes that showed a minimum of 12.5 % heterozygosity in the studied population of cattle, which stands for minimum 5 heterozygotes out of 40 genotyped animals. In our analyzed group of 29 genes, the lowest rate of heterozygotsity was 15 %, and the highest 67.5 %.

### Allelic expression imbalance

Out of 29 genes analyzed in three bovine tissues, 3 showed AEI: *LEP, IGF2, CCL2*. The rest of the genes showed no differences between allelic cDNA ratios and gDNA ratios or the cDNA ratios did not exceed the 60 %:40 % threshold.

**Fig. 2:**
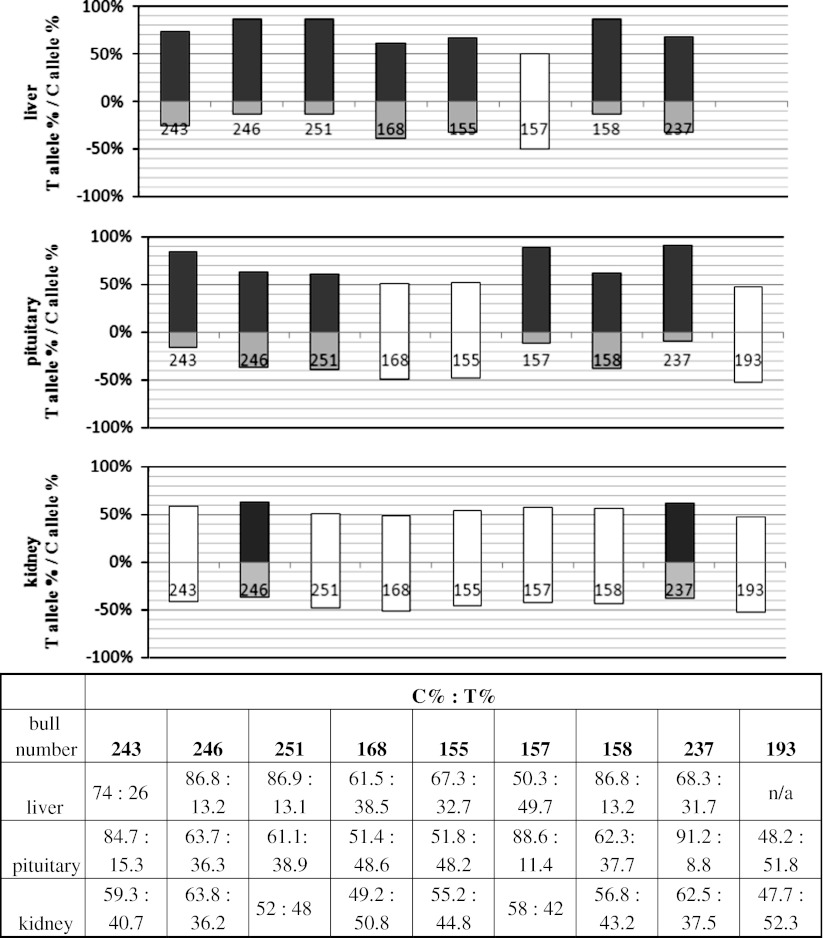
*LEP* allelic expression imbalance studied in liver, pituitary and kidney, showed as percentage of each allele in the allelic transcript pool. All of the samples have been standardized to allelic ratios measured in heterozygous gDNA. *Grey bars* represent imbalanced, and *white bars* represent balanced samples. Below the graphs the numerical presentation of results. Each bar is described with a bull number unique for each studied individual

For the *LEP* gene we chose the g.12140 C/T transition placed in exon 2 as the marker SNP. The gene showed allelic imbalance in all studied tissues. In liver, 7 out of 8 samples showed allelic imbalance, in pituitary 6 out of 9 and in kidney 2 out of 9 (Fig. 2). In all samples the C allele was over-expressed. In pituitary, liver and kidney the mean allelic ratios (±SD) were 5.39 (±3.64), 4.38 (±2.46) and 1.71 (±0.07), respectively. The mean ratio of all samples analyzed in liver was 3.68 and was significant at *p* < 0.05 (Table 2). Although the C allele was preferred the expression of this allele was never monoallelic.

**Table 2:** AEI mean ratios in studied tissues

Gene	Tissue	Mean allelic ratio^a^
*LEP*	Liver	3.68*
	Kidney	1.31
	Pituitary	3.50
*IGF2*	Liver	1.47
	Kidney	5.83***
	Pituitary	28.64***
*CCL2*	Kidney	1.33
	Pituitary	1.47

The columns represent the overall allelic ratio from all samples analyzed in a tissue, and only those samples that showed preferential expression towards one of the alleles

^a^The ratio was calculated from all samples analyzed in a tissue for a gene

* *p* < 0.05; *** *p* < 0.001

**Fig. 3:**
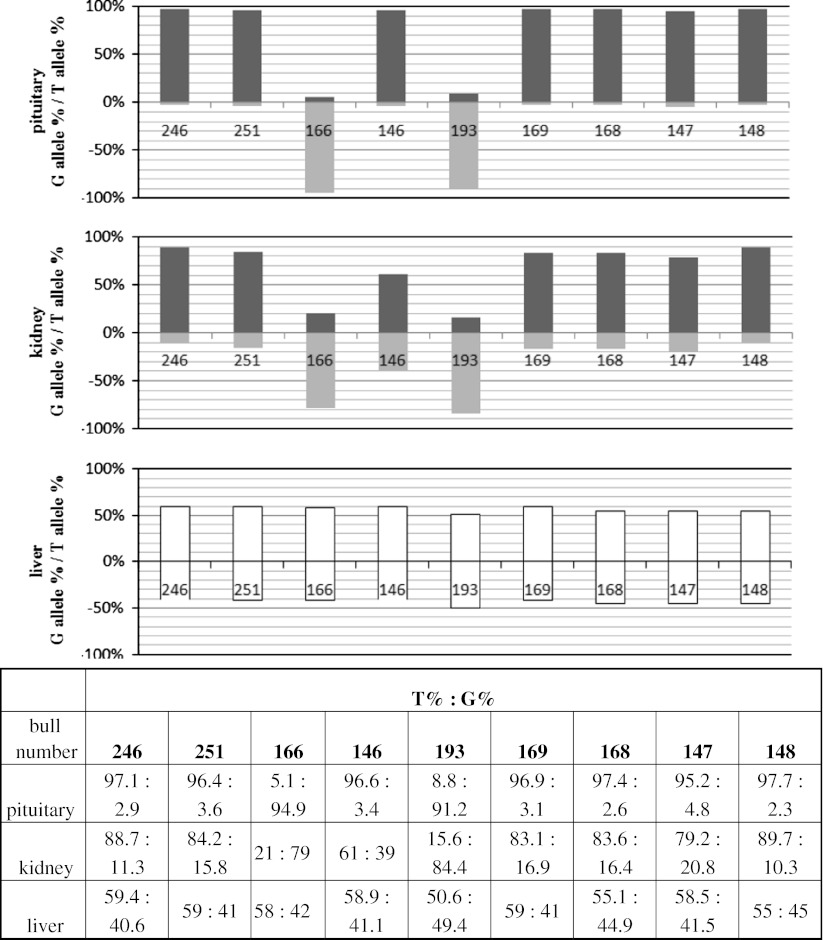
*IGF2* allelic expression imbalance studied in liver, pituitary and kidney, showed as percentage of each allele in the allelic transcript pool. All of the samples have been standardized to allelic ratios measured in heterozygous gDNA. *Grey bars* represent imbalanced, and *white bars* represent balanced samples. Below the graphs the numerical presentation of results. Each bar is described with a bull number unique for each studied individual

*IGF2* showed allelic imbalance in kidney and pituitary but not in liver. As the cSNP we used the g.24507 G/T transversion placed in exon 10. In each tissue 9 samples were analyzed. In pituitary 7 samples showed over-expression of the T allele and 2 of the G allele (Fig. 3). The mean ratio of allelic transcripts for the T allele over-expressed samples was 31.37 (±7.37) and for the G allele 15.67 (±10.1). Although both alleles were represented in over-expressed samples in pituitary the expression of the preferred variant was nearly monoallelic. In kidney, 7 samples indicated the T allele as the preferred in allelic expression and 2 indicated the G allele. The T allele showed a mean imbalance ratio of 4.51 (±0.12) and the G allele 5.94 (±1.88). The mean ratios of all samples analyzed in kidney and pituitary were 5.83 and 28.64 both significant at *p* < 0.001. No allelic imbalance was detected in liver.

**Fig. 4:**
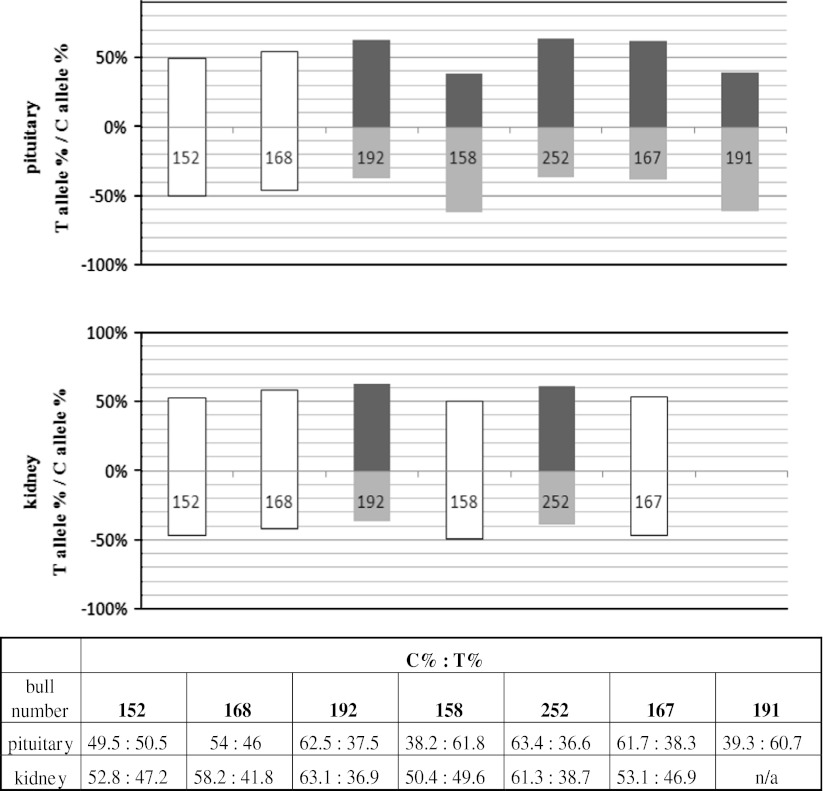
*CCL2* allelic expression imbalance studied in pituitary and kidney, showed as percentage of each allele in the allelic transcript pool. All of the samples have been standardized to allelic ratios measured in heterozygous gDNA. *Grey bars* represent imbalanced, and *white bars* represent balanced samples. Below the graphs the numerical presentation of results. Each bar is described with a bull number unique for each studied individual

For *CCL2* gene the c.249 C/T transition was used as the cSNP. *CCL2* transcript was not detected in liver and AEI was shown in pituitary and kidney (Fig. 4). In pituitary out of 7 samples, 3 showed C allele over-expression with a mean ratio of 1.67 (±0.06) and 2 samples showed T allele over-expression with a ratio of 1.58 (±0.05) but in kidney, only the C allele was over-expressed in 2 samples with the ratio of 1.65 (±0.08). Kidney and pituitary mean allelic ratios were 1.33 and 1.47, respectively.

**Fig. 5:**
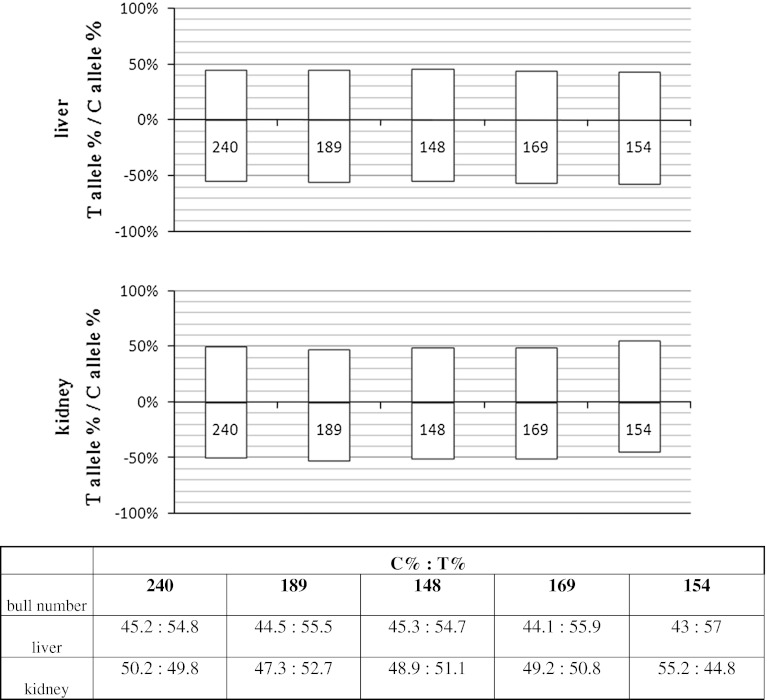
*PI* allelic expression imbalance studied in liver and kidney, showed as percentage of each allele in the allelic transcript pool. All of the samples have been standardized to allelic ratios measured in heterozygous gDNA. White bars represent balanced samples. Below the graphs the numerical presentation of results. Each bar is described with a bull number unique for each studied individual

*PI* gene was analyzed in all tissues used in this study with the c.989 C/T transition as a cSNP, but transcripts were detected only in liver and kidney (Fig. 5). cDNA from 5 individuals was analyzed in each tissue. None of them showed a deviation in allelic transcript proportions that would exceed the 60:40 threshold.

### Promoter analysis

The analysis of *LEP* gene 5′-flanking region (1,6 kb) indicated the presence of one CpG island (Supplementary Figure 1). According to Genome Browser the CpG region spans over part of intron 1, whole exon 1, and upstream of the transcription start site (TSS) and is 568 bp long and counting 41 CpG nucleotides. Compared to mouse and human the region close to the transcription initiation site is highly conserved between species. The human CpG island is 624 bp long and consists of 60 CpG dinucleotides whilst the murine is 215 bp long and consists of 17 CpGs. The analysis of polymorphic sites indicated the presence of 22 SNPs, and 8 putative transcription factor binding sites (TFBS) co-localizing with polymorphic sites (Supplementary Table 2).

The localization of promoters in bovine IGF2 was previously described [16], but the actual sequences are not known. Possible bovine IGF2 sequences were obtained by comparison with human IGF2 promoters. BLAST indicated sequences in the bovine sequence similar in 79, 71, 68 and 76 %, respectively for promoters 1, 2, 3 and 4. As the sequences derived from BLAST alignment sometimes had their end before or after the beginning of an exon, the search for SNPs and putative TFBS was limited to a beginning of an exon. The sequences analyzed were localized (according to GenBank accession EU518675): 18,038–18,709 bp for promoter 2; 19,918–20,535 bp for promoter 3; 22,033–22,555 bp for promoter 4. Our analysis indicated 3 SNPs in promoters 1–3, and no polymorphic changes in promoter 4. Out of these 3 SNPs only 2 of them harbor TFBS at polymorphic sites (Supplemental Table 3). Two CpG islands were detected in the putative promoter regions spanning from 18,553 to 20,266 bp and from 20,328 to 22,067 bp (according to GenBank accession EU518675).

The analysis of *CCL2* 3.0 kb 5′-flanking region indicated the presence of 8 SNPs, and 7 putative TFBS in polymorphic region (Supplementary Table 4). No CpG islands were detected in the analyzed region.

## Discussion

In this study we report an analysis of AEI presence in 29 randomly chosen genes expressed in at least one of the studied bovine tissues—liver, kidney or pituitary.

We chose to use pyrosequencing for the detection of AEI as it is more accurate in a quantitative approach, cheaper and less time-consuming comparing to standard Sanger sequencing and is recently often used for allele-specific gene expression studies (e.g. [43–45]). In contrast to Sanger sequencing, it is possible to automatically quantify the percentage of nucleotides incorporated at an indicated site—in this case the site of the observed mutation, and in consequence giving the proportion of allelic transcripts. Apart from negative controls applied before the PCR steps, it also has internal negative controls during the reaction. The detection of incorporated nucleotides is set to catch any sequence artifacts, as blank dispensations are also used in the analyzed sequence.

It is assumed that if a gene has a skewed allelic transcript ratio, it must be caused by a *cis*-factor influencing one of the alleles [1]. Many attempts have been made to connect AEI with a *cis*-factor [46, 47]. On the basis of human *IL13* allelic ratio a *cis*-factor responsible for the allelic ratio skew has been indicated 250 kb upstream of the gene [19].

In our experiment, out of the studied group, 3 genes: *IGF2*, *LEP* and *CCL2* showed AEI.

*LEP* showed AEI in all studied tissues. In samples showing the imbalance, the C allele was over-expressed relative to the T allele. The pattern of expression between tissues may indicate the causative factor does not show the same effect in different tissues, as the number of samples representing AEI and mean values of allelic transcript ratios were the highest in liver and the lowest in kidney.

Leptin is a 16 kDa protein secreted mainly in white adipose tissue. It has a major influence on food intake, energy balance. It plays an important role in processes critically dependent on energy supply such as reproduction and immune response [48].

Analysis of bovine leptin promoter showed that it is highly polymorphic—22 SNPs in a 1.6 kb 5′-flanking fragment of the gene ([49] and Supplemental Table 2). It has been indicated in our previous study that mutations in *LEP* promoter can influence gene expression, for example the C/G mutation at position −105 relative to transcription start site [50]. The CC genotype correlated with the highest gene expression in bovine liver and the GG with the lowest. The mutation is placed in the Sp1 transcription factor binding site and was proved to modulate Sp1 binding affinity to its target sequence hence influencing the transcription rate. Such a mutation is an example of a *cis*-regulatory factor. A thorough analysis of transcription factor binding sites in the bovine *LEP* promoter, indicated 8 binding sites for TFs that harbor SNPs in their motifs (including Sp1 factor at position −105) (Supplemental Table 2).

A comparative study of human, murine and bovine promoters indicated the presence of a CpG island (Supplementary Fig. 1). In bovine and human the CpG island overlapped a part of promoter, exon 1 and part of intron 1 of *LEP* gene. It is highly conserved between these species. It has been proven that certain CG dinucleotide methylation can modulate or silence *LEP* promoter activity in mice—one placed in the C/EBP binding motif and two in the vicinity of TATA-box [51]. Furthermore, a C/EBP putative binding site localized from −49 to −60 indicated by Liefers et al. [49], also has one CG nucleotide present which might indicate the presence of the same regulatory mechanism in cattle. The methylation density of the human *LEP* promoter is significantly higher when compared to the murine sequence (Supplementary Fig. 1). Also, what seems to be interesting, the patterns of methylation are acquired during postzygotic development [52]. In the bovine, the CpG island consists of 41 CG dinucleotides, four of which lie in polymorphic sites, and two of them in putative TFBS—Sp1 at position −105 and NF-1 at position −282 (Supplemental Table 2). Interestingly, at position −105, depending on the allele, the CG dinucleotide changes its position—allele C creates a CG dinucleotide at positions −104 and −105, and allele G at positions −105 and −106.

*IGF2* showed AEI in pituitary and kidney. In liver there were no found variations in allelic ratios. *IGF2* showed AEI regarding both studied alleles—G and T. In pituitary the mean ratios were so high that the expression of both variants could be nearly monoallelic. In kidney, mean allelic ratios were much lower comparing to pituitary, however all of the studied individuals showed AEI.

Insulin-like growth factor 2 (*IGF2*) is one of the best described genes in relation to its imprinting. In most embryonic and fetal tissues in human and other mammals the paternal allele is expressed and the maternal allele is silenced [53]. *IGF2* genetic structure is complex—it has four promoters activated in an age- and tissue- specific manner. Promoter-dependent transcripts are differentially spliced, thus also ranging in length. Promoter- and imprinting-dependent expression of *IGF2* has been compared between bovine fetuses, calves and adult bulls. In fetuses, as expected, paternal allele expression was monoallelic in all examined organs (liver, spleen, heart, bladder, lung, kidney and placenta) except for brain [16, 17]. In calves and bulls, the representation of maternal transcripts was increasing. In bull liver, expression was nearly biallelic, as it was shown in our previous study [18], which is consistent with the present results.

*CCL2* asymmetrical transcript expression was detected in both kidney and pituitary. In liver no *CCL2* transcript was detected or its quantity was so low that it was impossible to detect it with standard RT-PCR. Pituitary samples showed the highest mean of allelic transcript ratio. Both the C and T alleles were represented in samples showing allelic over-expression.

*CCL2* gene encodes small cytokines belonging to the CC chemokine family. These molecules and their receptors are likely to be responsible for leukocyte trafficking. Interactions between the receptors and ligands induce changes in monocytes and neutrophiles allowing cytosolic free Ca^2+^ to permit chemotaxis toward the inflammatory stimuli [54].

Our analysis of the 3.0 kb fragment upstream the transcription start site indicated 8 SNPs, 5 of which localize a putative TFBS (Supplemental Table 4). The 3.0 kb 5′-flanking region search for CpG islands did not show any specific CG concentrations as a potential target for methylation.

Therefore, the three genes showing AEI in this study are a possible representation of three different mechanisms causative for asymmetric allelic expression. *LEP* shows one-directional allele expression towards one prefereable allele, *IGF2* shows bidirectional expression in some cases ranging up to monoallelic expression (both alleles here are preferred), and *CCL2* indicates AEI only in few samples (both alleles are preferred) and the differences are not strong as compared to *LEP* and *IGF2.*

Our analysis of *PI* gene allelic expression showed biallelic balanced expression in all studied tissues. This differs from the previous findings regarding this gene [15] showing that in cattle fetal and dam kidney tissues some preferential monoallelic expression was shown. Although we used the same cSNP as a marker (c.989 C/T), the samples we used were derived from bulls, which might indicate the gender-specificity of allelic expression for this gene.

For the rest, of the analyzed 25 genes, there were no found evidence of AEI. Furthermore, there were no literature evidence indicating that allelic imbalance occurs in these genes. Thus, it is not proven that there is no such phenomena in their allelic ratio. Most of the genes were studied in 5 heterozygous individuals. It is possible that studying another heterozygous exonic SNP may have indicated an allelic imbalance in other individuals. Probably, a more global approach to the issue (e.g. transcritrome sequencing) would shed some light on the issue of AEI.

Summing up, this study as one of the first analyzed the phenomena of AEI on a larger group of genes in bovine. We showed that at least two autosomal genes (*LEP*, *CCL2*) not influenced by imprinting, showing an imbalance in the allelic transcript pool. Finding genes that have a skewed allelic ratio seems to be a helpful approach in the search of genetic factors that regulate gene expression and determine a phenotype. Such studies should be continued on a larger scale, as it is done in other species, by using, e.g. transcriptome sequencing to have a global insight to this phenomenon. Information on the animals’ pedigree and experiments on related animals, which would shed some light on the role parental-specific regulatory factors, would be very useful in this kind of study. Furthermore, although the genes for this study were chosen randomly, most of them are considered to be potentially important markers of production traits, thus possibly an analysis of their allelic ratios might be useful in animal breeding development.

## Electronic supplementary material

Below is the link to the electronic supplementary material.

Supplementary material 1 (DOCX 29 kb)

Supplementary material 2 (DOCX 13 kb)

Supplementary material 3 (DOCX 11 kb)

Supplementary material 4 (DOCX 11 kb)

Supplementary Fig. 1 *LEP* promoter analysis. (**a**) CpG island spanning from -421 bp to +148 bp relative to TSS—the island spans partly on the promoter sequence, exon 1 and partly intron 1 in the bovine *LEP* gene. (**b**) A comparison of human *LEP* promoter. The CpG island spans on a similar region like in cattle. Below, the conservation of mouse and bovine sequences relative to the human sequence
