# Supplementary material for: Allelic gene expression imbalance of bovine IGF2, LEP and CCL2 genes in liver, kidney and pituitary
Source: Mol Biol Rep. 2012 Nov 25;40(2):1189–200. doi: 10.1007/s11033-012-2161-3 (PMC3538019; doi:10.1007/s11033-012-2161-3)
Supplement: Supplementary file 1 — Supplementary material 1 (DOCX 29 kb) [file 11033_2012_2161_MOESM1_ESM.docx]

**Supplemental tab. 1** Primer sequences used for presequencing PCR and pyrosequencing (fwd – forward; rev – reverse; seq – sequencing). Primers with an M13 mark at their 5’ end had and additional sequence (5'-CGCCAGGGTTTTCCCAGTCACGAC-3') used for the 5’ biotinylated M13 universal primer . Number of samples for each tissue analyzed for this study was also added (n/d – gene expression was not detectable).

| **gene name** | **primer sequences 5'-3'** | | **annealing temperature**  **(°C)** | **amplicon length (bp)** | **number of samples analyzed** | | |
| --- | --- | --- | --- | --- | --- | --- | --- |
|  |  |  |  |  | **liver** | **kidney** | **pituitary** |
| ***CDH1*** | **fwd:** | AATGACCAAGAACGTGAGTCTCTG | **56** | **203** | **5** | **5** | **5** |
|  | **rev:** | M13 - ATGGCGGGAACTTGCAAT |  |  |  |  |  |
|  | **seq:** | GCAGGAAGGCACGGC |  |  |  |  |  |
| ***PVRL2*** | **fwd:** | TTCCACCCCCAGTATGGTC | **56** | **219** | **5** | **5** | **5** |
|  | **rev:** | M13 - CCAGGTGACTCCCCGACT |  |  |  |  |  |
|  | **seq:** | GCTGTCCTTCGTCAATAC |  |  |  |  |  |
| ***PPARGC1A*** | **fwd:** | M13 - TCCAAAACGGAAATACTCGTTAGT | **57** | **80** | **5** | **5** | **5** |
|  | **rev:** | GAGGAGGGGGCATCTTTATTTTCT |  |  |  |  |  |
|  | **seq:** | TTTTCTAGTTGTCTGGAGTC |  |  |  |  |  |
| ***FASN*** | **fwd:** | TGCAGGTCCTGGTGTCCA | **60** | **110** | **5** | **5** | **5** |
|  | **rev:** | M13 - CACGGCCAGGTTGAAGATG |  |  |  |  |  |
|  | **seq:** | CACCAGCGACGTCAG |  |  |  |  |  |
| ***CXCR2*** | **fwd:** | M13 - GTCCTGCCCCAGACCTTT | **55** | **181** | **5** | **n/d** | **n/d** |
|  | **rev:** | CGATCAGGACCAGGTTGTAGG |  |  |  |  |  |
|  | **seq:** | CATGGCCCGGTGCTT |  |  |  |  |  |
| ***CD14*** | **fwd:** | M13 - GCTCAGCGTGCTTGATCTCA | **57** | **136** | **5** | **n/d** | **n/d** |
|  | **rev:** | CGGGTCATTTTGGTGCTGG |  |  |  |  |  |
|  | **seq:** | GCAGCTCGTCTCGCC |  |  |  |  |  |
| ***MED28*** | **fwd:** | M13 - TCCAGAAACCAGAGCAAGTTATCA | **58** | **141** | **5** | **5** | **5** |
|  | **rev:** | TGTGCTGCATGTTGATGTCCT |  |  |  |  |  |
|  | **seq:** | TCGCTGTAATTCATTCC |  |  |  |  |  |
| ***FAM13A1*** | **fwd:** | ACTGAAGATATCGGAGGAGGAC | **55** | **123** | **5** | **5** | **5** |
|  | **rev:** | M13 - GCTTTATCCACCAGCTCTTGC |  |  |  |  |  |
|  | **seq:** | AGATATCGGAGGAGGAC |  |  |  |  |  |
| ***IBSP*** | **fwd:** | M13 - ACCACCGTTTGGGAAAATCAC | **58** | **117** | **5** | **5** | **5** |
|  | **rev:** | TAATTGTCCCCACGAGGATCT |  |  |  |  |  |
|  | **seq:** | TTGTTCATACTCCCCAG |  |  |  |  |  |
| ***PIT1*** | **fwd:** | M13 - ACCTGGAGAAAGAAGTGGTGAG | **53** | **180** | **n/d** | **n/d** | **5** |
|  | **rev:** | TGCATTCGAGATGCTCCTTAG |  |  |  |  |  |
|  | **seq:** | AATAGTAAATAAACTCTGAT |  |  |  |  |  |
| ***GHR*** | **fwd:** | M13 - GCGAGGTAGACGCCAAAAA | **54** | **115** | **5** | **5** | **5** |
|  | **rev:** | GTGGTAAGGCTTTCTGTGGTGAT |  |  |  |  |  |
|  | **seq:** | TGTCTTCCTGGTTAAAGC |  |  |  |  |  |
| ***PRL*** | **fwd:** | M13 - GCTCTTGGAATGACCCTCTGTA | **55** | **94** | **n/d** | **n/d** | **5** |
|  | **rev:** | TCAATCTCTATGGCCCTCGA |  |  |  |  |  |
|  | **seq:** | GGCTCCTTTCATACCC |  |  |  |  |  |
| ***IGF2*** | **fwd:** | M13 - CCAACCTCCCCACGTCAG | **59** | **69** | **9** | **9** | **9** |
|  | **rev:** | TTGAGGGGTTCATGATTGC |  |  |  |  |  |
|  | **seq:** | CCAGATGGAGGGGGC |  |  |  |  |  |
| ***GH*** | **fwd:** | TGCAGTTCCTCAGCAGAGTC | **55** | **78** | **n/d** | **n/d** | **5** |
|  | **rev:** | M13 - GGTCCTTCAGCTTCTCATAGACA |  |  |  |  |  |
|  | **seq:** | CAGAGTCTTCACCAACAG |  |  |  |  |  |
| ***ERB*** | **fwd:** | CTCACCTGCTGAATGCTGTGAC | **56** | **122** | **5** | **5** | **5** |
|  | **rev:** | M13 - TGCCTGACGTGAGACAGGA |  |  |  |  |  |
|  | **seq:** | TCTGGTCTGGGTGATT |  |  |  |  |  |
| ***PRNP*** | **fwd:** | M13 - ATCAACAGGGAGGCATTTAAAG | **55** | **185** | **5** | **5** | **5** |
|  | **rev:** | AAGTGCAAGCCAGTAATAACAGTG |  |  |  |  |  |
|  | **seq:** | GTCTATCTGCTAGGTGACA |  |  |  |  |  |
| ***ITGB5*** | **fwd:** | CGCTACGAAATGGCTTCAAAC | **55** | **101** | **5** | **5** | **5** |
|  | **rev:** | M13 - ACTGTGCCATTGTAGGATTTGTT |  |  |  |  |  |
|  | **seq:** | AACCCTCTGTACAGAAAGC |  |  |  |  |  |
| ***NOS2*** | **fwd:** | AATTGGGCAGCTATAGACGGTTC | **55** | **81** | **5** | **5** | **5** |
|  | **rev:** | M13 - ACTGCCTATGCAGGGTCTCGA |  |  |  |  |  |
|  | **seq:** | CAGCGTGAAGATCTCC |  |  |  |  |  |
| ***STAT1*** | **fwd:** | TTTTGGTATGGCTTTATGATGCT | **53** | **99** | **5** | **5** | **5** |
|  | **rev:** | M13 - ACTTTGGTATTAAAGCTGAAATGG |  |  |  |  |  |
|  | **seq:** | GGAAGTAAACTTTACAAATT |  |  |  |  |  |
| ***TNFa*** | **fwd:** | ATCAAGAGCCCTTGCCACAG | **57** | **137** | **5** | **5** | **5** |
|  | **rev:** | M13 - AGGTAGTCCGGCAGGTTGAT |  |  |  |  |  |
|  | **seq:** | AGGCCAAGCCCTGGT |  |  |  |  |  |
| ***LEP*** | **fwd:** | GCCCTATCTGTCTTACGTGGA | **60** | **106** | **8** | **9** | **9** |
|  | **rev:** | M13 - TGAGGGTTTTGGTGTCATCC |  |  |  |  |  |
|  | **seq:** | GAGGCTGTGCCCATC |  |  |  |  |  |
| ***SCD*** | **fwd:** | GATAAGCTGCCTACCTGAGTCAGT | **55** | **106** | **5** | **5** | **5** |
|  | **rev:** | M13 - GAACCTGCCTTTGCTTCTTG |  |  |  |  |  |
|  | **seq:** | GGCTTGCAGAAGCAGGG |  |  |  |  |  |
| ***PI*** | **fwd:** | ATATGCAAGTTCTGCCAATTTACA | **55** | **80** | **5** | **5** | **n/d** |
|  | **rev:** | M13 - CATCGCCCAGGACACTTTTTA |  |  |  |  |  |
|  | **seq:** | TGTCCATTTCTGAAACG |  |  |  |  |  |
| ***CCL2*** | **fwd:** | M13 - TGCAACATAGTATCTGCC | **54** | **70** | **n/d** | **6** | **7** |
|  | **rev:** | ATTTCTGCTTGGGGTCTGCAC |  |  |  |  |  |
|  | **seq:** | ATAACTCCTTGCCCAG |  |  |  |  |  |
| ***ODC1*** | **fwd:** | M13 - CAACCCAGCACTGGACAAGTAT | **55** | **91** | **5** | **5** | **5** |
|  | **rev:** | CGTGAAAGCTGATGCAACATAGTA |  |  |  |  |  |
|  | **seq:** | TGCAACATAGTATCTGCC |  |  |  |  |  |
| ***TGFb1*** | **fwd:** | AATGAAGTCTAGCTCGCACAGCA | **55** | **88** | **5** | **5** | **5** |
|  | **rev:** | M13 - CCGAGAGAGCAACACAGGTTC |  |  |  |  |  |
|  | **seq:** | TATATGTTCTTCAACACGTC |  |  |  |  |  |
| ***IL10RB*** | **fwd:** | M13 - TCCTCCGAAATCTTGAGTCAC | **55** | **74** | **5** | **5** | **5** |
|  | **rev:** | AGCTTTGTTCCGATCAGAAAGA |  |  |  |  |  |
|  | **seq:** | AAAGAAACCCTCGAACT |  |  |  |  |  |
| ***PSAP*** | **fwd:** | M13 - CCTAAGCCGAACATGTCTGCC | **55** | **117** | **5** | **5** | **5** |
|  | **rev:** | GTTGAGAGCGGAGCACACC |  |  |  |  |  |
|  | **seq:** | GAGCACACCTCCCCG |  |  |  |  |  |
| ***NOD2*** | **fwd:** | GCCCTTGAAAAGAATGACACC | **56** | **118** | **5** | **5** | **5** |
|  | **rev:** | M13 - CCTGGAAACATCAGAGCAAGAG |  |  |  |  |  |
|  | **seq:** | TTGAGAAACTCAGCCA |  |  |  |  |  |
