# Supplementary material for: Allelic gene expression imbalance of bovine IGF2, LEP and CCL2 genes in liver, kidney and pituitary
Source: Mol Biol Rep. 2012 Nov 25;40(2):1189–200. doi: 10.1007/s11033-012-2161-3 (PMC3538019; doi:10.1007/s11033-012-2161-3)
Supplement: Supplementary file 2 — Supplementary material 2 (DOCX 13 kb) [file 11033_2012_2161_MOESM2_ESM.docx]

**Supplementary tab 2.** Transcription factors that have putative binding sites at polymorphic sites indicated below in *LEP* promoter (GenBank accession number AJ571671). SNP positions are given relative to TSS. Bolded SNPs are placed in a CG nucleotide (one of the alleles creates a CG sequence) and are in the sequence indicated as a CpG island which spans from -421 bp to +148 bp relative to TSS. Additionally, the C/G SNP at position -105 creates two versions of CG dinucleotide – allele C creates a CG at positions -104 and -105, allele G creates a CG at positions -105 and -106.

| **SNP** | **position** | **TF** |  |
| --- | --- | --- | --- |
| **C/G** | **-105** | Sp1 |  |
| C/T | -147 | RPF1 |  |
| **C/T** | **-170** |  |  |
| G/A | -197 |  |  |
| **C/T** | **-201** |  |  |
| G/A | -211 | C/EBPalpha |  |
| G/A | -272 |  |  |
| **G/A** | **-282** | NF-1 |  |
| C/T | -292 | GR |  |
| -/G | -415 |  |  |
| G/A | -484 |  |  |
| G/C | -579 |  |  |
| A/T | -902 |  |  |
| C/T | -964 | Alx-4 |  |
| A/T | -1066 |  |  |
| -/G | -1198 |  |  |
| G/C | -1239 | TEF-1 |  |
| A/T | -1258 | c-Myb |  |
| G/A | -1391 |  |  |
| T/C | -1446 |  |  |
| A/G | -1452 |  |  |
| A/G | -1457 |  |  |
