# Supplementary material for: Allelic gene expression imbalance of bovine IGF2, LEP and CCL2 genes in liver, kidney and pituitary
Source: Mol Biol Rep. 2012 Nov 25;40(2):1189–200. doi: 10.1007/s11033-012-2161-3 (PMC3538019; doi:10.1007/s11033-012-2161-3)
Supplement: Supplementary file 3 — Supplementary material 3 (DOCX 11 kb) [file 11033_2012_2161_MOESM3_ESM.docx]

**Supplementary tab 3.** Transcription factors that have putative binding sites at polymorphic sites indicated below in *IGF2* possible promoter regions (promoters 1-3). SNP positions are given relative to TSS in exon 1.

^1 – SNP reported to be in the promoter 1 sequence^

^2 - SNP reported to be in the promoter 2 sequence^

^3 - SNP reported to be in the promoter 3 sequence^

| **SNP** | **position** | **TF** |
| --- | --- | --- |
| C/T^1^ | -204 |  |
| C/G^2^ | +16714 | NF-1 |
| A/C^3^ | +17689 | c-MYC, USF2, NF-k, Sp1 |
