# Supplementary material for: Allelic gene expression imbalance of bovine IGF2, LEP and CCL2 genes in liver, kidney and pituitary
Source: Mol Biol Rep. 2012 Nov 25;40(2):1189–200. doi: 10.1007/s11033-012-2161-3 (PMC3538019; doi:10.1007/s11033-012-2161-3)
Supplement: Supplementary file 4 — Supplementary material 4 (DOCX 11 kb) [file 11033_2012_2161_MOESM4_ESM.docx]

| **SNP** | **position** | **TF** |
| --- | --- | --- |
| G/A | -112 | POU1F1a, LCR-F1 |
| T/- | -691 |  |
| C/A | -1371 | p53, Meis-1a |
| C/T | -1807 | delta factor |
| C/T | -2570 |  |
| G/A | -2692 | GR |
| G/A | -2769 | Sp1 |
| G/A | -2781 |  |

**Supplementary tab 4.** Transcription factors that have putative binding sites at polymorphic sites indicated below in *CCL2* 5’-flanking region (3,000 bp upstream of CCL2 transcription start site) SNP positions are given relative to TSS.
