# Supplementary figures and images for: Allelic gene expression imbalance of bovine IGF2, LEP and CCL2 genes in liver, kidney and pituitary
Source: Mol Biol Rep. 2012 Nov 25;40(2):1189–200. doi: 10.1007/s11033-012-2161-3 (PMC3538019; doi:10.1007/s11033-012-2161-3)

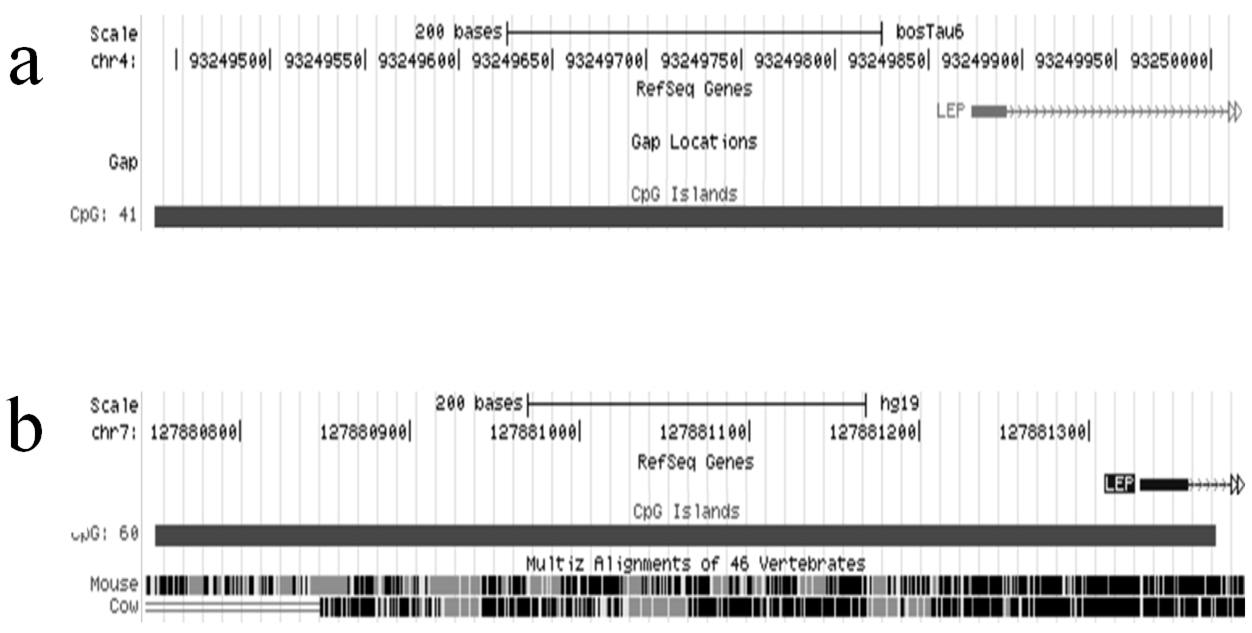

Supplement: Supplementary file 5 — LEP promoter analysis. (a) CpG island spanning from -421 bp to +148 bp relative to TSS—the island spans partly on the promoter sequence, exon 1 and partly intron 1 in the bovine LEP gene. (b) A comparison of human LEP promoter. The CpG island spans on a similar region like in cattle. Below, the conservation of mouse and bovine sequences relative to the human sequence [file 11033_2012_2161_MOESM5_ESM.jpg]
